# Medical Gossip

**Published:** 1861-04

**Authors:** 


### Art. XVI.—MEDICAL GOSSIP.

The recently published volumes, in which Professor Owen has given to the world the posthumous manuscripts of John Hunter, are fruitful sources of medical gossip. They have more than a merely literary interest. They recal the whole of that strange history which attached to the literary remains of Hunter. Although known to many in its main features, the lapse of time has effaced the recollection and the details of that singular and chequered story, and to the younger men it is only dimly and vaguely known. The whole chain of events is drawn before the eye by this publication, which adds some last and most important links. The manuscript remains of Hunter included a magnificent body of notes on the Animal and Vegetable Kingdom, physiological observations, records of many hundred dissections, scattered materials for great works on Comparative Anatomy, on Natural History, on Psychology, Geology, and on special subjects, such as the History of Monsters, and the Structure and Composition of Animal bodies. Until the year 1800 the Hunterian collections and manuscripts were the property of John Hunter's executors. At the beginning of the year 1800 the collections having been purchased by Parliament, were transferred to the custody of the Royal College of Surgeons, then the Corporation of Surgeons. The manuscripts were, by Sir Everard Home's order, carted away to his house, and were not allowed to accompany the collections. They consisted of masses of original notes, which Mr. Hunter had laboriously written or dictated to Mr. Clift and Mr. Bell, his amanuenses, during thirty years of his life. Here were the recorded notes of his dissections of many hundreds of mammalia, birds, reptiles, fishes, insects, and invertebrate animals. All these Sir Everard Home transferred to his house in the year 1800. He kept them there, and continued to publish papers in the *Transactions of the Royal Society*, and subsequently a work in four volumes on Comparative Anatomy, in which he ran over the whole ground which Hunter had arduously cultivated during his life. Then in 1823, when the last sheets of the fourth volume of his book were corrected, he destroyed these manuscripts by fire, and in doing so he nearly set fire to his house. This conduct he vindicated before a Committee of the House of Commons, by asserting that these papers were desired by Hunter to be shielded from the public eye, and that he having used them as far as possible for the benefit of the collections in the Museum of the College of Surgeons, had felt it his duty to destroy them. A more sinister motive has always been more than suspected. It is now painfully but providentially made manifest.

The reverent care of Clift, the curator of Hunter's museum in Leicester Fields, had led him, during the six years after Hunter's death, when he kept long watch over the collection in Leicester Fields, and when "he had, as he might say, no other books to read," to make careful copies of almost one-half of these manuscripts, and copious extracts from others. He could not bring himself during his life, or during the life of his unworthy relative, Sir Everard Home, to publish these precious transcripts, which Home in his published works pillaged wholesale. But he carefully used them for the illustration of the Hunterian collections; and, scattered through all the volumes of the catalogues, are precious fragments of transcripts which he furnished to Professor Owen, in elucidation of intricate series of preparations. Finally, at his death he bequeathed these MSS., on which he had spent much time and care, to Professor Owen. That distinguished anatomist, who has devoted so many years of his life to the study, the development, and the exposition of the Hunterian collections, has now given these valuable papers to the world, enriched with notes minutely referring each paragraph to the preparation, when existing, to which it relates, carefully and judiciously annotating the rough manuscripts where actually necessary, and, in every case, determining, with the marvellous analytical skill which he eminently possesses, the species and genus of the subjects from the rough indications of these notes. One only of the posthumous papers is wanting here. It is that on Fossils. The private history of this manuscript is hardly less scandalous than that of the others. This MS. was not destroyed by Sir Everard Home, and was presented by his son to the College in April, 1839. Professor Owen had cognizance of this paper, and, in February, 1855, suggested to the Council of the College of Surgeons that it should be published with the catalogue of Hunterian fossils then in preparation. In March, 1855, he delivered three lectures on the Hunterian fossils, in which he read this MS. in the theatre of the College, and elegantly expounded its nature and importance. All this produced no effect on the Council. In October, 1859, Professor Owen being employed in preparing the press copies of Mr. Clift's manuscript for the present volumes, applied to the Council for permission to include that MS. in the collection. Strange, indeed, it is to have to say, that permission was refused. But the long-neglected manuscript was forthwith hurried to the press, and, having been edited with a lamentable ignorance and inefficiency, was published within eight weeks. The editor, who had done nothing to elucidate the text, did, however, contrive to inflict an additional disgrace on the College; for, in the preface, he falsely asserted that the MS. had not been brought to notice by the Hunterian Professor (Mr. Owen) until after the publication of the last volume of the Catalogue of Fossils, when he had unexpectedly (in 1856) read it from the chair. Thus, by postponing for a year the date of Professor Owen's lecture, he gave colour to an insinuation that the Hunterian Professor had been guilty of suppressing for a time this manuscript. The indignant protest of Professor Owen, when challenged by the *Lancet* as to the truth of this charge, will be remembered. Its consequence was, that the Council of the College had to retract the charge, and to expunge the odious falsehood from the printed preface. It has been the fate of these papers to be treated by those who have best profited by the fertility and greatness of Hunter, with the most unexampled neglect, perfidy, and illiberal meanness. It has, on the other hand, been their fortune to have been studied and appreciated by true men, such as Clift and Owen, who reverenced Hunter, who entered in a kindred spirit into his labours, and who have finally rescued them from destruction and from oblivion by their reverent and devoted care. This chapter of literary history closes with the publication of the present volumes, and the scientific, medical, and literary world will feel that Mr. Owen has, in no slight degree, added to his claims on their respect and gratitude by the part which he has played in endowing them with this admirable edition of the posthumous papers of the great English naturalist and surgeon.

The faults of individual members of the Council, of course, can never affect the honour of the whole body, and these facts make a sad and pitiful contrast with the eloquent lip-worship which Mr. Coulson, a few weeks since, offered to the memory of Hunter, in the philosophic "Hunterian Oration," which he delivered in the theatre of the College.

The lamented death of Dr. Baly, the learned, upright, and philosophic attendant on the Queen, has rendered vacant during the quarter one of the highest appointments which a physician can, in this country, be called to fill. Speculation as to his successor pointed to Dr. Acland, of Oxford, who had accompanied the Prince of Wales to Canada, Dr. T. K. Chambers, who had acted as physician to the Prince of Wales on his visit to Rome, and Dr. Gull. It is believed that the appointment was actually offered to Dr. Acland, whose high accomplishments, great erudition, and perfect knowledge of the world in all its best phases, have procured for him well-deserved favour at Court. But as Regius Professor at a great seat of learning, and possessing a freedom and an influence which Court attendance could only shackle and diminish, that physician had little to gain by the exchange. The appointment of Dr. Jenner was somewhat unexpected. But it is an appointment with which the profession are, and have reason to be, well pleased. Dr. Jenner's reputation is founded upon long and legitimate labour in the field of strictly medical observation, which he has pursued with quiet but undeviating energy, His clinical labours are known throughout Europe, and his appointment is a tribute to quiet worth and laborious research. The appointment was, of course, subject to the advice of Sir James Clark, whose failing strength has rendered it necessary. We rejoice to hear that his present condition is one of improved health.

It were greatly to be wished, that of Sir Benjamin Brodie, his colleague in courtly office, words of cheerful import might also be spoken. But the sight of that distinguished chieftain of surgery is still veiled with a thick mist, which successive operations for glaucoma and cataract, following each other at short intervals, have not served to dispel. The painful circumstances attending these operations will be regretted by all, and scarcely less so the indelicacy with which rumours and counter rumours, and puffs and counter puffs were publicly circulated, more or less at variance with truth, and intended apparently to protect or assail the reputation of the operator rather than to allay the useless fears or encourage the just hopes of the friends of the great surgeon.

The ill-health and advanced age of Mr. Lloyd, one of the veteran and perennial surgeons of St. Bartholomew's Hospital, has made a vacancy in the staff at that institution. Mr. Wormald will be elected in his place. We behold here, therefore, the spectacle of a grey-haired and distinguished man, who, after thirty years of hospital service as an assistant, is now, at the age of threescore, advanced for the first time to the post of surgeon. In the autumn of his life, and at a time when his reputation is established, when his prime is already past, when his energies are damped, and when, in all professions and employments, men look to enjoy the fruits of a life of labour, he is invited to advance the career of usefulness open to a man newly appointed to the care of beds in a great hospital, and he is told to begin when he should be already at the end.

At the same moment the committee of another metropolitan hospital, St. Mary's, Paddington, have decided that at that institution thenceforth, the surgeon in charge of in-patients shall not hold office for a longer term than fifteen years, or after sixty years of age. This provision is in accordance with the dictates of justice and wisdom. After some ten years or more of service as surgeon in charge of out-patients, and fifteen as surgeon in charge of in-patients, every reasonable man must feel that he has done all for the hospital which can be expected from him, and that the hospital has done all for him which he ought to expect from it. The tenacity of the veterans who cling to their accustomed haunts, and grasp the reins until they fairly fall from their feeble hands, is comprehensible and is pardonable, but it is impolitic and unjust.

---

